# Taxonomic and Functional Metagenomic Profile of Sediment From a Commercial Catfish Pond in Mississippi

**DOI:** 10.3389/fmicb.2018.02855

**Published:** 2018-11-22

**Authors:** Seong Won Nho, Hossam Abdelhamed, Debarati Paul, Seongbin Park, Michael J. Mauel, Attila Karsi, Mark L. Lawrence

**Affiliations:** ^1^Department of Basic Sciences, College of Veterinary Medicine, Mississippi State University, Starkville, MS, United States; ^2^Amity Institute of Biotechnology, Amity University, Noida, India; ^3^Department of Animal and Dairy Sciences, Mississippi State University, Starkville, MS, United States

**Keywords:** metagenome, sediment, aquaculture pond, catfish, eutrophic, nitrogen metabolism, sulfur metabolism, methanogenesis

## Abstract

Metagenomic analyses of microbial communities from aquatic sediments are relatively few, and there are no reported metagenomic studies on sediment from inland ponds used for aquaculture. Catfish ponds in the southeastern U.S. are eutrophic systems. They are fertilized to enhance algae growth and encourage natural food production, and catfish are fed with commercial feed from spring to fall. As result, catfish pond sediment (CPS) contains a very dense, diverse microbial community that has significant effects on the physiochemical parameters of pond dynamics. Here we conducted an in-depth metagenomic analysis of the taxonomic and metabolic capabilities of a catfish pond sediment microbiome from a southeastern U.S. aquaculture farm in Mississippi using Illumina next-generation sequencing. A total of 3.3 Gbp of sequence was obtained, 25,491,518 of which encoded predicted protein features. The pond sediment was dominated by *Proteobacteria* sequences, followed by *Bacteroidetes*, *Firmicutes*, *Chloroflexi*, and *Actinobacteria*. Enzyme pathways for methane metabolism/methanogenesis, denitrification, and sulfate reduction appeared nearly complete in the pond sediment metagenome profile. In particular, a large number of *Deltaproteobacteria* sequences and genes encoding anaerobic functional enzymes were found. This is the first study to characterize a catfish pond sediment microbiome, and it is expected to be useful for characterizing specific changes in microbial flora in response to production practices. It will also provide insight into the taxonomic diversity and metabolic capabilities of microbial communities in aquaculture. Furthermore, comparison with other environments (i.e., river and marine sediments) will reveal habitat-specific characteristics and adaptations caused by differences in nutrients, vegetation, and environmental stresses.

## Introduction

Channel catfish production is the largest aquaculture industry in the United States with total sales of $380 million in 2017 (USDA, 2018). Aquaculture is one of the most rapidly growing food production sectors (Food Agriculture Organization of the United Nations and Fisheries and Aquaculture, 2014), but it faces several production and environmental challenges to maintain its economic viability (Schreier et al., 2010). Fish production ponds are rich in dissolved nutrients due to intensive feeding and fecal waste. Unconsumed feed, fish feces, and senescent phytoplankton are deposited into aquaculture sediments, which can enhance the microbial flora in the sediments and lead to anoxic conditions (Holmer and Kristensen, 1992). Medicated feed, such as Terramycin (oxytetracycline), Romet-30 (sulfadimethoxine-ormetoprim), and Aquaflor (florfenicol), as well as fertilizers may also impact pond sediment microflora. In turn, changes in sediment microflora impact fish health in aquaculture systems.

Fish excrete nitrogen in the form of ammonia, which is toxic to fish; nitrification by chemolithoautotrophic bacteria in pond sediments is an important process that prevents toxic buildup. The microbes contributing to nitrification consist of two functional groups: the ammonia-oxidizing bacteria (AOB) and ammonia-oxidizing archaea (AOA) that convert toxic ammonia to nitrite, and the nitrite-oxidizing bacteria (NOB) that oxidize nitrite to the less toxic nitrate. AOB include the genera *Nitrosomonas*, *Nitrosococcus*, and *Nitrosospira* (Kowalchuk and Stephen, 2001). AOA include *Nitrosopumilus* from marine water (Konneke et al., 2005). The NOB group includes genera *Nitrobacter, Nitrospira*, and *Nitrospina* (Wang et al., 2014). Pond sediments also have potential to serve as a reservoir for fish pathogens. Therefore, it is important to understand microbial flora in aquaculture pond sediments because of its impact on the pond ecosystem, especially in nutrient cycling, concentrations of organic and inorganic nutrients and toxins, and effects on fish health.

Metagenomic analysis allows assessment of mixed environmental microbial communities by directly sequencing DNA from environmental samples (Dinsdale et al., 2008). This approach provides a picture of the diversity and microbiome structure present in the environment (Simon and Daniel, 2009), and it enables studies to understand how microbial diversity is modulated in response to environmental or anthropogenic impacts (Larsen et al., 2012). Thus, it is particularly appropriate for assessing the taxonomic and functional microbial diversity in a pond sediment biome and monitoring community changes over space and time (Nogales et al., 2011).

Previous culture-independent studies investigating sediment microbial phylogenetic structure showed that microbial communities are indicators of both the physicochemical status of freshwater sediments (Logue et al., 2008; Gibbons et al., 2014) and ecological degradation (Feris et al., 2009). A few metagenomes have been published from deep-sea sediment (Kimes et al., 2013), mangrove (Andreote et al., 2012), and river systems (Staley et al., 2013), and recent studies have examined the response of fish gut-associated microbial communities from aquaculture systems in response to lifestyle and dietary preference (Wu et al., 2010; Xing et al., 2013). Signatures of bacterial composition were found in shrimp farming (Sousa et al., 2006), and pyrosequencing was used to explore bacterial diversity and detect potential fish pathogens during production of *Scophthalmus maximus* (turbot) and *Solea senegalensis* (sole) (Martins et al., 2013).

In pond sediments, the microbiome is critical for maintenance of homeostasis conditions, including toxin removal and cycling of carbon, nitrogen, and phosphorus (Brock, 1970; Leung et al., 1994). In particular, nitrogen is very important in aquaculture as a nutrient and potential toxicant, and it is an essential requirement for phytoplankton and bacterial growth in anaerobic conditions. In the current study, we present a description of the microbiome found in sediment from a catfish research pond maintained under commercial production conditions. Our results are culture-independent and based on metagenomic sequence of total DNA extracted directly from the pond sediment and analyzed by Illumina sequencing. We describe the microbial taxa present in catfish pond sediment and the potential metabolic processes that appear to be occurring in this ecosystem affecting carbon, nitrogen, and sulfur cycling. This analysis also provided a fundamental baseline profile of the catfish pond sediment microbiome for comparison with sediments from other environments.

## Materials and Methods

### Sediment Sampling, DNA Extraction, and Sequencing

Sediment sampling was performed in October 2012 (water temperature approximately 20°C) in sediments from a 0.8-hectare aquaculture research pond stocked with approximately 6,000–8,000 catfish (*Ictalurus punctatus*) with average size of 0.5 kg at the Delta Research and Extension Center, Stoneville, MS, United States. The pond was maintained at typical stocking and feeding parameters used for catfish aquaculture. Three samples were collected at 10 am using a modification of a previously published method (Schneegurt et al., 2003). In brief, ∼500 g per sample of pond sediment was collected at 5–10 cm of sediment depth ∼8 m from the pond bank in a water depth of ∼1.2 m using a sterile spatula and immediately transferred to sterile 50 ml tubes kept on ice.

Genomic DNA was extracted from each sediment sample separately using MoBio PowerSoil DNA Isolation Kit (Mo Bio Laboratories, Carlsbad, CA, United States) according to manufacturer’s protocol with 5.0 g of sediment per extraction. A NanoDrop (Thermo Scientific, Wilmington, DE, United States) spectrometer was used to quantify extracted DNA and to assess DNA quality. Illumina library preparation and sequencing followed manufacturer’s protocols (Illumina, San Diego, CA, United States). Briefly, genomic DNA was sheared by sonication and separated by electrophoresis on a 1% agarose gel. Gel slices corresponding to ∼300 and ∼500 bp were excised and purified using QIAquick Gel Extraction Kit (Qiagen). The blunt-ended DNA fragments were A-tailed using the Quick Blunting Kit (New England BioLabs) and purified. Sheared DNA was ligated to sequence adapters. All ligated libraries were enriched using standard PCR (11–12 cycles) with Illumina paired end primers before library quantification and validation. One microgram DNA was used in library construction. The amplified libraries were pooled in an equimolar ratio and sequenced using an Illumina HiSeq 2000 (Illumina, San Diego, CA, United States) using paired end reads of 100 bases. Raw reads containing three or more “N” bases or contaminated by adapter (>15 bp overlap) were removed by Trimmomatic (Bolger et al., 2014), and the filtered clean reads were used for metagenomic analysis.

### Taxonomic Distribution and Functional Analysis of Metagenomic Sequence

The taxonomic analysis was performed using BLASTX against the SEED and Pfam databases (Altschul et al., 1997) on the MG-RAST server^1^ using a cut-off *E*-value of 1e-5, minimum identity of 60%, and a minimum alignment length of 15 bp (Meyer et al., 2008). BLASTX was also conducted using MetaGenome Analyzer software (MEGAN v5) with the lowest common ancestor (LCA) algorithm used to visualize results (Huson et al., 2007). Using BLASTX and BLASTN, reads were compared against the NR and NT NCBI databases. Analysis was performed comparing distinct hierarchical levels, and a directed homogeneity test was used to identify significant differences in sample comparisons. Multiple testing correction analysis was not applied, and all unassigned reads were ignored.

Statistical analysis was performed using results from the MG-RAST annotation system, and results were visualized using Statistical Analyses of Metagenomic Profiles (STAMP) (Parks and Beiko, 2010) to detect biologically relevant differences in the relative proportion of sequences. Paired metagenomic samples were used for the analysis, and statistical significance of the differences between samples was assessed by the Two-sided Fisher’s Exact test. Story’s false discovery rate (FDR) was used for multiple test correction as recommended by STAMP. Results with *q*-value (<0.05) were considered significant, and unclassified reads were removed from the analysis.

Functional classification was conducted using BLASTX (cut-off *E*-value of 1e-5) against COGs (Tatusov et al., 2001), which was downloaded from hierarchical classification in MG-RAST server using NCBI database. BLASTX and subsystem analysis were used against the SEED-NR database in MG-RAST for functional sequence annotation with the same parameters as a taxonomic distribution. A functional analysis using the SEED (Overbeek et al., 2005) and KEGG (Kanehisa et al., 2004) databases was conducted using MG-RAST sever. Each sequence was associated with its SEED functional role using the best BLAST score to protein sequences without known functional roles. A similar procedure was used to match each sequence to a KEGG orthology (KO) accession number. Results were matched with each protein’s RefSeq database, and relative abundance was used to identify enzymes in important metabolic pathways. Matches with alignment scores higher than 80 were retained.

To the best of our knowledge, there is no previous information about structure and function of bacterial communities in aquaculture pond sediments. Therefore, the microbial community from aquaculture sediment was compared to freshwater sediment from the Tongue River in Southeastern Montana (MG-RAST ID 4481977.3) (Gibbons et al., 2014) and deep-sea sediment of the Gulf of Mexico (MG-RAST ID 4465489.3) (Kimes et al., 2013). A classification was used to determine the sample that most closely clustered to the taxonomic composition or metabolic potential of the sediment metagenome (*E*-value of 1e-5, the minimum identity of 60%, and a minimum alignment length of 15 bp).

## Results and Discussion

### Sequence Generation

Whole community microbial DNA from catfish pond sediment (CPS) was sequenced, making this study the first metagenomic survey of a catfish aquaculture environment (MG-RAST ID 4583113.3). Of the 29,278,265 sequences (totaling 2,927,826,500 bps) that passed quality control, 3,690,631 sequences (11.2% of total) were identified as artificial duplicate reads (ADRs), which are nearly identical sequences that result from sequencing two or more copies of the exact DNA fragment (Niu et al., 2010). Of the sequences without rRNA genes, 26,127,903 contained predicted protein features, 4,424,138 (16.9%) of which were assigned an annotation using at least one protein database, and 21,703,765 (82.9% of features) contain predicted proteins with unknown function. A total of 2,855,527 sequences (64.5% of annotated proteins) were assigned to functional categories (Supplementary Table S1). At the domain level, Bacteria (96.8%) dominated, while the Archaea (1.2%) and the Eukaryota (1.5%) contributed substantially less to the CPS community.

### Taxonomic Profiles

The numbers of sequences affiliated with each bacterial taxon in CPS were similar with other sediment samples, with a dominance of *Proteobacteria* (46.0%) and an abundance of *Bacteroidetes* (15.9%), *Firmicutes* (8.6%), *Chlorflexi* (5.1%), and *Actinobacteria* (5.0%) (Table 1). Minor groups represented at the phylum level included *Cyanobacteria*, *Verrucomicrobia*, *Planctomycetes*, *Chlorobi*, and *Acidobacteria*. The four most abundant classes of bacteria were *Deltaproteobacteria*, *Bacteroidia*, *Clostridia*, and *Actinobacteria* according to MG-RAST analysis (Table 1).

**Table 1:** Bacterial classifications and abundance in the CPS metagenome.

Taxonomic group	Number of reads	Abundance (%)	Number of genera
*Proteobacteria* (phylum)	2,971,341	46.0	
*Deltaproteobacteria*	1,408,697	21,8	66
*Betaproteobacteria*	596,878	9.2	87
*Gammaproteobacteria*	501,097	7.8	165
*Alphaproteobacteria*	415,93	6.4	119
*Epsilonproteobacteria*	36,824	0.6	13
*Bacteriodetes* (phylum)	1,027,970	15.9	
*Bacteroidia*	415,272	6.2	8
*Flavobacteriia*	254,455	3.9	29
*Cytophagia*	184,562	2.9	10
*Spingobacteriia*	130,637	2.0	6
*Firmicutes* (phylum)	557,836	8.6	
*Clostridia*	360,863	5.6	70
*Bacilli*	167,236	2.6	43
*Chloroflexi* (phylum)	331,544	5.1	
*Chloroflexi*	132,079	2.1	4
*Actinobacteria* (phylum)	326,195	5.0	
*Actinobacteria*	326,195	5.0	106


**FIGURE 1:**
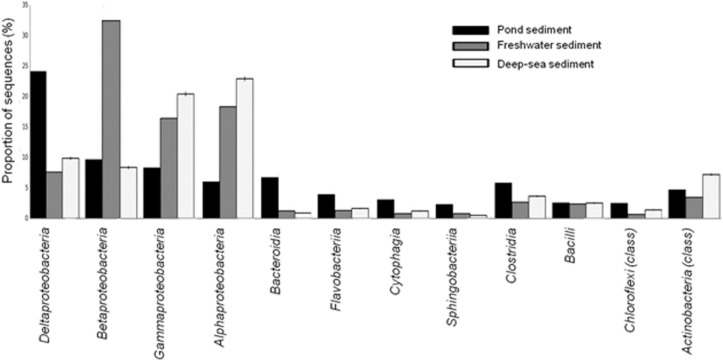
Taxonomic distribution of bacterial classes from the CPS and other aquatic sediments from freshwater and deep-sea environments.

The *Proteobacteria* associated with each sample were examined more closely to evaluate the potential of both aerobic and anaerobic biodegradation. In aquaculture ponds and lakes, the top sediment layer down to a few millimeters is typically aerobic, but below this depth sediment is anaerobic (Boyd and Tucker, 1998). The high occurrence of *Deltaproteobacteria* in CPS, which is not commonly observed in metagenomes from water or sediment samples (Figure 1), might be related to the catfish habitat, where eutrophic and anaerobic conditions could drive selection for specific microbial groups such as sulfate associated bacteria (Harrison et al., 2009). The *Deltaproteobacteria* in our sample was mostly comprised of a branch of strictly anaerobic genera containing many of the known sulfate- and sulfur-reducing bacteria including *Desulfovibrio*, *Desulfobacter*, *Desulfococcus*, *Desulfonema*, and *Desulfuromonas* spp. High organic loads and anaerobic conditions in pond sediments yield ideal conditions for sulfate reduction and sulfide production (Boyd and Tucker, 1998). Many of the *Deltaproteobacteria* were also species involved in methane transformation, which was paralleled by the presence of other bacteria with anaerobic physiology such as ferric iron-reducing *Geobacter* spp. (Supplementary Table S2).

*Deltaproteobacteria*, in particular, are capable of fumarate addition to both aromatic and aliphatic hydrocarbons, hence activating the anaerobic hydrocarbon biodegradation pathway. The increases in *Deltaproteobacteria* correlated with an increase in other protein-coding genes involved in anaerobic degradation of hydrocarbons, such as benzylsuccinate synthase (BSS), acetyl-CoA acetyltransferase, and benzoyl-CoA reductase (Kimes et al., 2013). Anthropogenic hydrocarbon loading from catfish feed may enrich for microbial species with anaerobic hydrocarbon degradation capabilities. Other types of *Proteobacteria*, including *Beta-*, *Gamma-*, and *Alphaproteobacteria* had relatively lower representation compared with microbial populations from other aquatic sediments from river or deep sea environments (Figure 1). Moreover, the CPS had a higher prevalence of *Epsilonproteobacteria* than the river and deep-sea sediments. *Epsilonproteobacteria* are prevalent in the digestive tracts of animals and serve as symbionts or pathogens; their energy metabolism involves oxidizing reduced sulfur, formate, or hydrogen coupled with the reduction of nitrate or oxygen (Takai et al., 2005).

The phylum *Bacteroidetes* is very diverse and includes *Cytophaga*, *Flexibacter*, and *Bacteroides* (Woese, 1987; Woese et al., 1990). The *Bacteroidetes* phylum is comprised of four classes: *Bacteroidia*, *Flavobacteria*, *Cytophage*, and *Sphingobacteria*, which include around 7,000 different species (Bergey et al., 2011). The *Bacteroidetes* phylum in CPS included the *Flavobacteria*, which has many aquatic species (Table 1), and it also contained opportunistic human pathogens (Bernardet and Nakagawa, 2006), including the genera *Elizabethkingia*, *Weeksella* and *Capnocytophaga* (Kim et al., 2005; Leadbetter, 2006) (Supplementary Table S2). *F. psychrophilum*, *F. columnare*, and *F. branchiophilum* are some *Bacteroidetes* species that have economic impacts on freshwater fish, causing infections that can have severe effects on farmed and wild fish (Hawke and Thune, 1992; Loch and Faisal, 2015). *Flavobacterium* infections were first reported a century ago in aquaria (Davis, 1922).

Previously, *Firmicutes* was observed as a dominant phylum in the digestive tract of many marine and freshwater fish species (Austin, 2006; Wu et al., 2012). In the present study, *Firmicutes* was found to be prevalent in CPS. In particular, *Clostridium* sp. was the most abundant genus within *Firmicutes* and represented 5.6% of the identified sequences (Table 1), making it more abundant in CPS compared to marine and river sediments (Figure 1). *Clostridium* sp. are commonly found in human and animal guts and can form endospores, allowing survival under unfavorable environments (Davies et al., 1995; Mueller-Spitz et al., 2010). *Clostridium* sp. contribute to hydrolytic enzyme production, suggesting a possible role in degradation of organic matter. In eutrophic marine cage aquaculture sediments, metabolism is dominated by anaerobic decomposition (Holmer and Kristensen, 1992); therefore, enrichment of *Clostridium* sp. may be a good indicator of the impact of organic matter in aquatic sediments. Interestingly, some lactic acid bacteria were detected in the *Firmicutes* phylum in CPS, including the genera *Lactococcus*, *Streptococcus*, and *Enterococcus* (Supplementary Table S2). Lactic acid bacteria are generally considered to be non-pathogenic (Ringø and Gatesoupe, 1998). However, some species including *Lactococcus garvieae*, *Streptococcus shiloi*, and *Streptococcus difficile* were reported as fish pathogens (Eldar et al., 1994, 1996).

**FIGURE 2:**
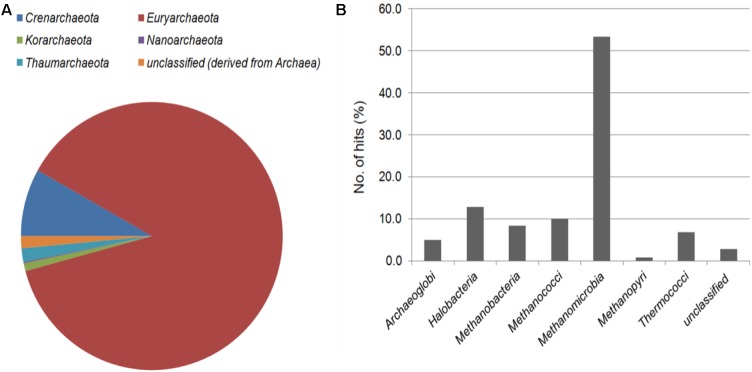
Taxonomic affiliation of archaeal reads in the CPS metagenome at the phylum **(A)** and class **(B)** level.

The highest proportion of archaeal reads within the CPS metagenome was *Euryarchaeota* (87.6%), which was composed of several classes: *Archaeoglobi* (1,159 reads), *Halobacteria* (3,001 reads), *Methanobacteria* (1,956 reads), *Methanococci* (2,334 reads), *Methanomicrobia* (12,467 reads), and *Thermococci* (1,593 reads) (Figure 2). *Methanosarcina* was the most abundant genus and accounted for 31% of the total archaeal sequences. This genus includes many methanogens involved in both acetotrophic and hydrogenotrophic methanogenesis, which is a type of anaerobic respiration that generates methane and is considered the terminal step in organic decomposition. Most *Methanosarcina* are non-motile and mesophilic, and they are unique among archaeal bacteria in that most can utilize multiple substrates as electron acceptors, including methanol (Kandler and Hippe, 1977). Thus, *Methanosarcina* is among the most adaptable and flexible of the methanogens (Maeder et al., 2006).

**FIGURE 3:**
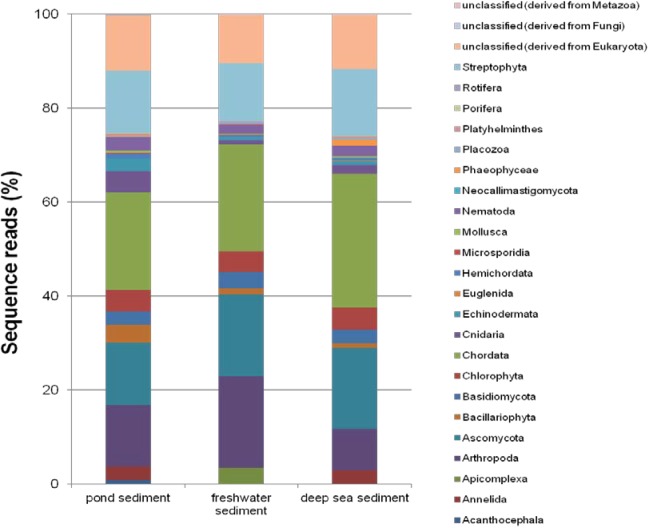
Comparison of the relative abundance of eukaryotic reads within the 23 represented phyla in CPS and two previously published marine sediments.

The eukaryotic sequences represented 23 phyla from the *Animalia*, *Fungi*, *Plantae*, and *Protista*. The *Animalia* phylum *Chordata* (20.76 – 28.52%) showed predominant abundance in the three sediments, followed by *Athropoda*, *Ascomycota*, and *Steptophyta*. *Chordata* was the most abundant in deep-sea sediment, but the *Arthropoda* was lower compared to CPS and river sediment. The *Bacillariophyta*, *Cnidaria*, and *Echinodermata* phyla had greater abundance in CPS compared to river sediment, and *Apicomplexa* was in greater abundance in river sediment (Figure 3).

### Functional Categories

**FIGURE 4:**
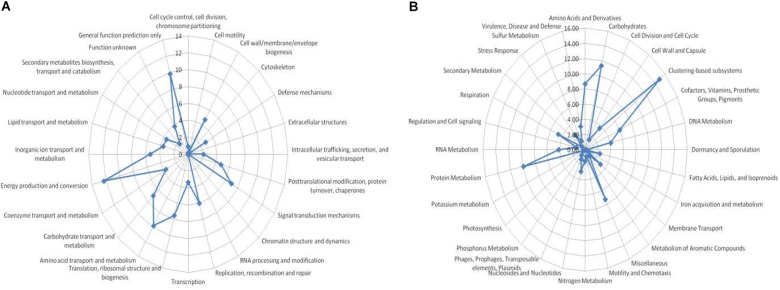
Functional assignment of metagenome sequences. **(A)** BLASTX analysis against the COGs database: percent was assigned to specific COG functional categories, and **(B)** BLASTX analysis against GenBank conducted using MG-RAST; percent abundance was assigned to specific KEGG identifiers.

Environmental DNA from CPS had matches in 24 COG and 28 KEGG functional categories, respectively (Supplementary Table S3). The dominant COG functions were prokaryotic, with high abundance of sequence reads in energy production and conversion as well as amino acid transport and metabolism. In particular, CPS had a high number of sequences in signal transduction mechanisms, carbohydrate transport and metabolism, inorganic ion transport and metabolism, and general function prediction. A lower percent of reads was found for functions associated with eukaryotic organisms (RNA procession and modification, chromatin structure and dynamics, cell motility, and cytoskeleton and extracellular structures) (Figure 4A). The most abundant KEGG functional categories were carbohydrate metabolism, clustering-based subsystems, miscellaneous, amino acids and derivatives, and protein metabolism (Figure 4B).

**FIGURE 5:**
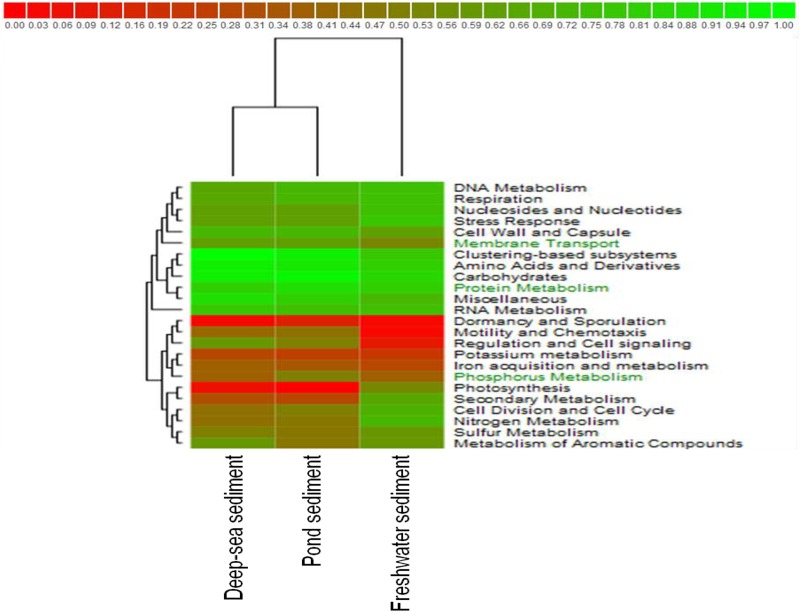
Hierarchical clustering combined with heat mapping based on functional subsystem classifications for CPS, freshwater sediment of the Tongue River in Southeastern Montana, and deep-sea sediment of the Gulf of Mexico.

The metabolic potential of the CPS metagenome was compared with two freshwater and deep-sea sediment metagenomes publicly available on the MG-RAST server. A heat map showed that the CPS metagenome is similar to the deep-sea sediment metagenome. CPS had more sequences within phosphorous metabolism, protein metabolism, and membrane transport than the other aquatic sediments (Figure 5). Phosphorus is relatively abundant in catfish ponds because producers often fertilize ponds to encourage algal blooms, and protein is also relatively abundant due to application of commercial feeds.

Based on our taxonomic analysis, we expected to detect genes encoding enzymes involved in methanogenesis, which is considered the final step in decomposition. As expected, we detected methanogenesis genes encoding an F420-dependent *N*(5),*N*(10)-methylenetetrahydromethanopterin reductase (EC 1.5.99.11), *N*(5),*N*(10)-methenyltetrahydromethanopterin cyclohydrolase (EC 3.5.4.27), formylmethanofuran dehydrogenase (EC1.2.99.5), CoB–CoM heterodisulfide reductase (EC1.8.98.1), coenzyme F420 hydrogenase (EC 1.12.98.1), N5-methyltetrahydromethanopterin (EC 2.1.1.86), and the enzyme responsible for the last step of methanogenesis, methyl coenzyme M reductase (EC 2.8.4.1) (Table 2).

**Table 2:** Numbers of represented gene variants in the CPS metagenome for different functions.

Metabolism Methane	Function	No of sequence reads	No of genes
Particulate methane monooxygenase (EC 1.14.13.25)	Oxidation of ammonia, methane, halogenated hydrocarbons, and aromatic molecules	20	11
Methanol dehydrogenase (EC 1.1.99.8)	Methanol to formaldehyde.	271	41
*S*-formylglutathione hydrolase (EC 3.1.2.12)	*S*-formylglutathione to formic acid and glutathione	73	44
F420-dependent reductase (EC 1.5.99.11)	CO_2_ to methane	209	25
*N*(5), *N*(10)-methenyltetrahydromethanopterin cyclohydrolase (EC 3.5.4.27)	CO2 to methane	30	12
Formylmethanofuran dehydrogenase (EC1.2.99.5)	CO2 and methanofuran to *N*-formylmethanofuran.	229	77
CoB–CoM heterodisulfide reductase (EC1.8.98.1)	Reduction of the heterodisulfide of the methanogenic thiol-coenzymes, coenzyme M, and coenzyme B	5,015	274
Coenzyme F420 hydrogenase (EC 1.12.98.1)	CO_2_ to methane	59	22
*N5*-methyltetrahydromethanopterin (EC 2.1.1.86)	Transfer of the methyl group from *N5*- methyltetrahydromethanopterin to coenzyme M	83	44
Methyl-coenzyme M reductase (EC 2.8.4.1)	Methyl-coenzyme M and coenzyme B to methane (anaerobic oxidation).	73	25
Nitrogen			
Nitrogenase (EC 1.18.6.1)	Nitrogen to ammonia	2,226	172
Nitrate reductase (EC 1.7.99.4)	Nitrate to nitrite	3,573	456
Nitrite reductase (1.7.1.4, EC 1.7.7.1 and 1.7.2.1)	Reduction of nitrite	1,517	270
Cytochrome c552 precursor (EC 1.7.2.2)	Nitrite to ammonia	2,418	104
Nitric-oxide reductase (EC 1.7.99.7)	Nitric oxide to nitrous oxide	3,067	134
Nitrous-oxide reductase (EC 1.7.99.6)	Nitrous oxide to dinitrogen	1,027	49
Hydroxylamine reductase (EC 1.7.3.4)	Hydroxylamine to ammonia and water	4	3
Sulfur			
Sulfate adenylyltransferase (EC 2.7.7.4)	Transfer of the adenylyl group from ATP to inorganic sulfate, generating adenosine 5′-phosphosulfate and pyrophosphate.	7,133	884
Adenylyl sulfate kinase (EC 2.7.1.25)	Catalyze the synthesis of activated sulfate	2,144	276
Phosphoadenylyl sulfate reductase (EC 1.8.4.8)	Reduction of activated sulfate into sulfite	413	122
Adenylyl sulfate reductase (EC 1.8.99.2)	Adenosine 5′-phosphosulfate (APS) to sulfite and AMP	1,186	50
Sulfite reductase (EC 1.8.99.1, 1.8.1.2 and 1.8.7.1)	Sulfite to sulfide	1,559	305
Other			
Oxidase stress catalase (EC 1.11.1.6)	Hydrogen peroxide to water and oxygen	5,846	454
Peroxidase (EC 1.11.1.7)	Oxidation of organic compounds	5.183	269


**FIGURE 6:**
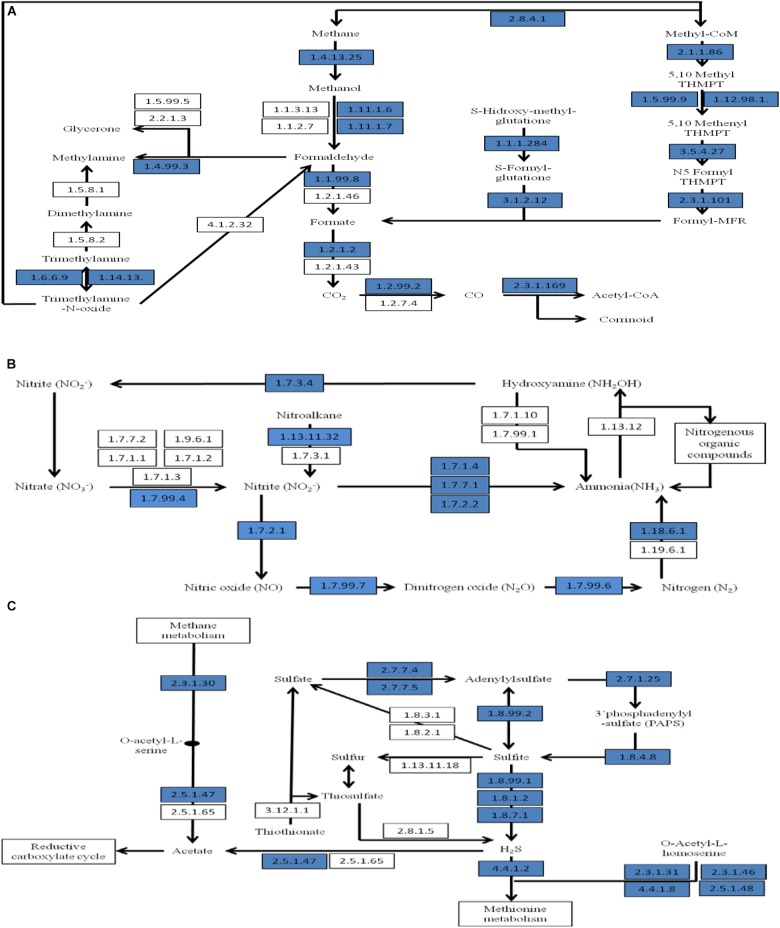
Part of a SEED-based functional analysis of the CPS metagenome. **(A)** Carbon fixation and methane metabolism; **(B)** nitrogen metabolism; and **(C)** sulfur metabolism. Blue boxes are proteins that were represented in CPS.

The transformation of methane and methanol into formaldehyde and then formate in CPS was suggested by metabolic reconstruction, mainly from the activity of particulate methane monooxygenase (EC 1.14.13.25), methanol dehydrogenase (EC 1.1.99.8), and *S*-formylglutathione hydrolase (EC 3.1.2.12). We also detected genes encoding enzymes involved in formaldehyde fixation, including genes for enzymes that incorporate methane into organic compounds via the serine pathway or the ribulose monophosphate pathway, indicating that the CPS microbiome is able to metabolize methane as their source of carbon and energy to survive. Compared to the metagenome of freshwater sediment from the Tongue River, both CPS and the freshwater sediment microbiomes encode processes for formaldehyde fixation; the distinct sediments differ only in the particular pathways used. In CPS, methane metabolism is encoded by the *Gammaproteobacteria* using the ribulose monophosphate pathway to assimilate carbon; in addition, part of the *Alphaproteobacteria* utilized the serine pathway of carbon assimilation. Oxidation of formate yields carbon dioxide, and a high abundance of genes encoding proteins involved in the conversion of carbon dioxide into carbon monoxide and later into acetyl-CoA was detected (Figure 6A). Formate also contributes to oxidative stress response; oxidase stress catalase (EC 1.11.1.6) and peroxidase (EC 1.11.1.7) were detected in CPS, both of which are antioxidant enzymes that contribute to limiting oxidative damage by reactive oxgen species (ROS) such as H_2_O_2_ (Table 2).

Analysis of nitrogen metabolism revealed genes encoding nitrogen immobilization and mineralization in CPS (Figure 6B). Sequences encoding nitrogen fixation were detected, including atmospheric nitrogen fixation using a nitrogenase (EC 1.18.6.1) that converts nitrogen gas to ammonia. A high abundance of nitrification species was detected in the metagenome (Supplementary Table S3) such as *Nitrosomonas* spp., *Nitrobacter* spp., and *Nitrococcus* spp. These species oxidize ammonia to nitrate, preventing toxic buildup of ammonia that can affect fish health. Genes encoding nitrification such as hydroxylamine oxidoreductase (EC 1.7.3.4) for oxidation of hydroxylamine were found. Genes encoding denitrification enzymes, including nitrate reductase (EC 1.7.99.4), nitrite reductase (EC 1.7.1.4, EC 1.7.7.1 and 1.7.2.1), cytochrome c552 precursor (EC 1.7.2.2), nitric-oxide reductase (EC 1.7.99.7) and nitrous-oxide reductase (EC 1.7.99.6) were observed (Table 2). Denitrification is the dissimilatory reduction of nitrate into nitric oxide, dinitrogen oxide, and nitrogen. The balance among these pathways is affected greatly by environmental conditions including oxygenation, temperature, nitrate concentration, and organic matter content in the sediment (Saunders and Kalff, 2001).

The predominant type of sulfur metabolism encoded in the CPS generates the reductive form of sulfite and hydrogen sulfide (H_2_S) (Figure 6C). Most of genes observed were involved in conversion of sulfate into adenylylsulfate and to sulfite and H_2_S, including sulfate adenylyltransferase (EC 2.7.7.4), adenylylsulfate kinase (EC 2.7.1.25), phosphoadenylyl sulfate reductase (EC 1.8.4.8), adenylylsulfate reductase (EC 1.8.99.2), and sulfite reductase (EC 1.8.99.1, 1.8.1.2 and 1.8.7.1) (Table 2). Enzymes mediating reduction of sulfate and adenylylsulfate into H_2_S dominated sulfur metabolism in CPS (Figure 6C). Generated H_2_S can influence the reductive carboxylate cycle (CO_2_ assimilation) and might be released by volatilization, producing the typical smell of mangrove swamps (Andreote et al., 2012). Sulfate-reducing bacteria obtain energy by oxidizing organic matter or hydrogen using sulfate (or other sulfur molecules) as electron acceptors, yielding H_2_S. They are prevalent in environments such as swamps and standing waters that have low oxygen. Sulfur-reducing bacteria and some archaea are similar, but they use elemental sulfur as an electron acceptor, and they also produce H_2_S. During catabolism of organic matter, hydrogen sulfide is also released by other anaerobic bacteria when sulfur-containing amino acids are digested. In CPS, *Deltaproteobacteria* was the most abundant, possibly indicating the importance of sulfate reduction in this environment (Wrighton et al., 2014).

Overall, the metabolism of carbon, nitrogen, and sulfur are interlinked within the microbial population, and this is particularly true for the metabolism of sulfur and carbon. The abundance of organic matter in the anaerobic environment of CPS yields an optimal environment for several anaerobic bacteria such as sulfate-reducing bacteria and methanogens (Dar et al., 2008). These groups share similar environmental niches, and their relative abundance is controlled by substrate availability (Oremland and Polcin, 1982). Simple substrates are important for methanogens, while sulfate-reducing bacteria are capable of degrading more complex substrates, including long chain and aromatic hydrocarbons (Muyzer and Stams, 2008).

Genes encoding resistance to antibiotics and toxic compounds (RATC) represents a subset of virulence genes that made up 3.45% of the classified metagenome in CPS. By comparison, genes encoding RATC proteins generally make up ∼2–2.24% of the classified metagenome in other aquatic ecosystems. Compared to other aquatic sediments, the CPS metagenome encoded a higher proportion of proteins in copper homeostasis, cobalt-zinc-cadmium resistance, multidrug resistance efflux pumps, and resistance to fluoroquinolones; however, the CPS metagenome encoded a lower proportion of arsenic resistance, beta-lactamase, erythromycin resistance, methicillin resistance, and resistance to vancomycin (Table 3). In particular, genes encoding cobalt-zinc-cadmium energy-dependent efflux pump had the highest proportion in the RATC category in CPS. By contrast, the fish gut microbiome encodes a much higher proportion of mercury resistance, mercuric reductase, and cobalt-zinc-cadmium genes than other metagenomes (Durso et al., 2012). Fluoroquinolone is of particular interest regarding the use of antibiotics in animal agriculture. Two enzymes are the principal targets for the antibacterial activity of fluoroquinolone: DNA gyrase and topoisomerase IV, which introduce negative supercoiling and prevent the accumulation of excess supercoiling (Collignon and Angulo, 2006). The DNA gyrase subunit B gene and topoisomerase IV subunit A were most frequently associated with *Clostridia*, *Actinobacteria*, *Bacteroidetes* and with *Proteobacteria* in the CPS metagenome, but whether they carry mutations associated with resistance is not currently known. Beta-lactamase genes were most frequently associated with *Alpha*- and *Gammaproteobacteria*, which encode resistance to beta-lactam antibiotics (penicillins and cephalosporins). The true risk to public health from antimicrobial use and subsequent resistance in aquaculture is speculative. However, the presence of antibiotic resistance genes and elements in pond sediments is a threat to public health if the resistance genes are transferrable to clinically significant pathogens.

**Table 3:** Proteins in the CPS metagenome in the resistance to antibiotics and toxic compounds (RATC) category.

Function	No. of sequences^∗^	No. of hits^†^
Adaptation to D-cysteine (catalyze the transformation of D-cysteine into pyruvate, H_2_S, and NH_3_)	63	41
Aminoglycoside adenylyltransferases (confers resistance to kanamycin, gentamicin, and tobramycin)	13	12
Arsenic resistance	5,506	598
Beta-lactamase (inactivates beta-lactam antibiotics including penicillins and cephalosportins)	5,912	1112
Bile hydrolysis	97	47
BlaR1 family regulatory sensor (controls expression of beta-lactamase)	9,705	1,257
Cadmium resistance	195	65
Cobalt-zinc-cadmium resistance	58,584	3,252
Copper homeostasis	12,481	1,788
Erythromycin resistance	352	147
Mercury resistance operon	360	67
Methicillin resistance in Staphylococci	3,029	656
MexE-MexF-OprN multidrug efflux system	499	67
Multidrug resistance efflux pumps	23,910	1,881
Resistance to vancomycin	129	64
Resistance to chromium compounds	519	106
Resistance to fluoroquinolones	21,073	1,744
The mdtABCD multidrug resistance cluster	954	204
Zinc resistance	5,771	204


*^∗^The number of sequences that contain a given annotation.*^†^*No. of hits refer to the number of unique database sequences that were found in the similarity search without double counting.*

Of these RATC categories, beta-lactamase resistance, multidrug resistance efflux pumps, fluoroquinolone resistance, cobalt/zinc/cadmium resistance, and acriflavine resistance genes are present in other metagenomes such as lake sediment, soil, feces, and marine environment (Durso et al., 2012). This broad distribution across agricultural, environmental, and human-associated samples indicates that these mechanisms are generally distributed and suggests they are functionally important in diverse habitats.

## Conclusion

Catfish pond sediment is a highly eutrophic, nutrient-rich, and diverse ecosystem that has significant effects on the physiochemical parameters of pond dynamics. This study is a pacesetting metagenomics analysis using Illumina sequencing for sediment in an intensive aquaculture system, and it revealed significant coupling between phylogeny and functional potential. The community structure suggests that the distribution of particular taxa is driven by their metabolic capabilities in response to the environment. For example, *Deltaproteobacteria* was the most abundant class, which is unique in the CPS metagenome compared to other aquatic sediments, and it demonstrates capability of anaerobic hydrocarbon metabolism. Functionally, our analysis revealed that the metagenome likely has significant impacts on nitrogen, phosphorus, and sulfur dynamics in catfish production ponds.

Further work to assess pond sediment could yield a complete description of the potential metabolic pathways in the CPS metagenome. The current work establishes a critical baseline for the CPS metagenome for comparison with other distinct environments. Also, future work could assess the effects of environmental changes (for example, feeding changes or antimicrobial use) in catfish production ponds on the sediment metagenome.

## Author Contributions

ML and MM supervised the study. ML, MM, and AK designed the experiments. HA, SN, SP, DP, and MM performed the experiments. SN, SP, and ML analyzed and interpreted the data. All authors wrote and approved the manuscript.

## Conflict of Interest Statement

The authors declare that the research was conducted in the absence of any commercial or financial relationships that could be construed as a potential conflict of interest.

## Footnotes

1 http://metagenomics.anl.gov/

## Supplementary Material

Table S1 Sequence data from the CPS metagenome.

Table S2 Functional profiles (COG and KEGG) for genes identified in the CPS metagenome.

Table S3 Relative abundances of bacteria by systematic classifications within the CPS metagenome.
