# Supplementary material for: Taxonomic and Functional Metagenomic Profile of Sediment From a Commercial Catfish Pond in Mississippi
Source: Front Microbiol. 2018 Nov 22;9:2855. doi: 10.3389/fmicb.2018.02855 (PMC6262407; doi:10.3389/fmicb.2018.02855)
Supplement: Table S1 — Sequence data from the CPS metagenome. [file Table_1.DOCX]

**TABLE S1 |** Sequence data from the CPS metagenome

| **Parameter** | **Value** |
| --- | --- |
| Total sequence bp count | 3,303,312,400 |
| Artificial duplicate reads sequence count | 3,690,631 |
| Post QC bp count | 2,927,826,500 |
| Sequence length | 100 ± 0 bp |
| GC percent | 56.14 ± 14 % |
| Predicted protein features | 26,127,903 |
| Predicted rRNA features | 311,695 |
| Identified protein features | 4,424,138 |
| Identified rRNA features | 12,533 |
| Identified functional categories | 2,855,527 |
